# Development of 3D-printed myoelectric hand orthosis for patients with spinal cord injury

**DOI:** 10.1186/s12984-019-0633-6

**Published:** 2019-12-30

**Authors:** Hyun-Joon Yoo, Sangbaek Lee, Jongheon Kim, Chanki Park, Boreom Lee

**Affiliations:** 1 Department of Biomedical Science and Engineering (BMSE), Institute Integrated Technology (IIT), Gwangju Institute of Science and Technology (GIST), 123 Cheomdan-gwagiro, Buk-gu, Gwangju, 61005 South Korea; 2 Department of Mechanical Engineering, Inha University, 100, Inha-ro, Michuhol-gu, Incheon, 22212 South Korea; 3 School of Mechanical Engineering, Gwangju Institute of Science and Technology (GIST), 123 Cheomdan-gwagiro, Buk-gu, Gwangju, 61005 South Korea

**Keywords:** Spinal cord injury, Assistive wearable robots, Orthosis, Three-dimensional printing

## Abstract

**Background:**

Spinal cord injury (SCI) is a severe medical condition affecting the hand and locomotor function. New medical technologies, including various wearable devices, as well as rehabilitation treatments are being developed to enhance hand function in patients with SCI. As three-dimensional (3D) printing has the advantage of being able to produce low-cost personalized devices, there is a growing appeal to apply this technology to rehabilitation equipment in conjunction with scientific advances. In this study, we proposed a novel 3D-printed hand orthosis that is controlled by electromyography (EMG) signals. The orthosis was designed to aid the grasping function for patients with cervical SCI. We applied this hand exoskeleton system to individuals with tetraplegia due to SCI and validated its effectiveness.

**Methods:**

The 3D architecture of the device was designed using computer-aided design software and printed with a polylactic acid filament. The dynamic hand orthosis enhanced the tenodesis grip to provide sufficient grasping function. The root mean square of the EMG signal was used as the input for controlling the device. Ten subjects with hand weakness due to chronic cervical SCI were enrolled in this study, and their hand function was assessed before and after wearing the orthosis. The Toronto Rehabilitation Institute Hand Function Test (TRI-HFT) was used as the primary outcome measure. Furthermore, improvements in functional independence in daily living and device usability were evaluated.

**Results:**

The newly developed orthosis improved hand function of subjects, as determined using the TRI-HFT (*p* < 0.05). Furthermore, participants obtained immediate functionality on eating after wearing the orthosis. Moreover, most participants were satisfied with the device as determined by the usability test. There were no side effects associated with the experiment.

**Conclusions:**

The 3D-printed myoelectric hand orthosis was intuitive, easy to use, and showed positive effects in its ability to handle objects encountered in daily life. This study proved that combining simple EMG-based control strategies and 3D printing techniques was feasible and promising in rehabilitation engineering.

**Trial registration:**

Clinical Research Information Service (CRiS), Republic of Korea. KCT0003995. Registered 2 May 2019 - Retrospectively registered.

## Background

Spinal cord injury (SCI) is a major disorder that causes sensory, motor, and autonomic dysfunction in parts of the body served by the spinal cord below the level of injury. It often leads to permanent sequelae and eventually results in extensive physical and emotional disabilities in patients, families, and society [1]. Although the epidemiology of the SCI demonstrates some variation between countries, the overall incidence is suspected to be 23.0 cases per million worldwide [2]. Unfortunately, there is no treatment to completely recover the functional status for patients with SCI at present [3]. As a result, concerns about disability and medical costs due to SCI have been growing worldwide in both developed and developing countries [4]. In particular, cervical SCI is a more serious situation than thoracic or lumbar SCI because it is accompanied by other medical disorders such as autonomic or respiratory dysfunction [5, 6]. Moreover, cervical SCI affects upper extremity function, which is the most crucial consequence for tetraplegic patients. Therefore, most patients with cervical SCI expect a considerable improvement in quality of life would occur if their hand function improved [7]. In this respect, rehabilitation of hand function is paramount for cervical SCI.

Even a small improvement in hand function may lead to an increase in functional independence in daily living for people with tetraplegia. Therefore, various rehabilitation strategies have been developed to maximize the recovery of hand function in tetraplegia. Patients with cervical SCI have reportedly undergone neuromuscular electrical stimulation represented by functional electrical stimulation as well as conventional rehabilitation exercise including occupational therapy [8, 9]. However, despite these treatments, SCI still remains an incurable disease and there is little chance of recovery for patients with complete SCI [10]. Surgical options such as nerve transfer have been tried to restore hand function; however, studies are in the early stages with only a small number of cases [11, 12]. Recently, new alternative therapeutic options such as robotic rehabilitation [13] or neural stem cell transplantation have been actively developed. In a recent study, allogenic neural stem cell transplantation in 12 patients with chronic SCI showed some promising results. However, the study is still in the preclinical stage [14].

Despite advances in medical knowledge and treatment of SCI, it is true that most of the current treatment options aim to minimize secondary complications and utilize the residual functions. Therefore, orthoses have been a mainstay of SCI rehabilitation and many individuals with hand paralysis utilize hand orthoses to compensate their physical function. Various hand orthoses have been developed to assist patients with cervical SCI with hand disabilities. However, most orthoses are rigid molded plastic and used to correct hand deformities or reduce contractures and have limitations with regards to providing functional movements [15]. Although one of the conventional metal tenodesis splints, the flexor hinge splint can be used to promote prehension grip, most patients rarely use the splint due to its cost and design. The Rehabilitation Institute of Chicago plastic tenodesis splint can be an alternative solution because it is easier to fabricate and is relatively cheap. However, it can only be applied to patients who are capable of wrist extension against gravity [16]. Furthermore, while new robotic exoskeletons are being developed, externally powered hand orthoses are rarely commercialized due to their high cost and poor aesthetics. An electrically-powered functional hand orthosis has been commercialized by Broadened Horizons, Minnesota, USA (PowerGrip), but the cost is more than $5000 [17].

Three-dimensional (3D) printing is a computer-aided manufacturing method that can create 3D objects using various materials such as plastic, metal, liquids, or even living cells [18, 19]. Since this technology is beneficial with regards to cost-effectiveness, customization, and enhanced productivity, it has attracted much interest in the biomedical field [20, 21]. It was first applied to make dental implants in the early 2000s. Since then, the biomedical applications of the technology have evolved considerably into various categories, including rehabilitation engineering [22]. Furthermore, the application of 3D printing technology to the development of new orthoses is thought to be a promising avenue for cost reduction with rapid manufacturing compared to previous metal orthoses. Currently, many publications reference the use of 3D printing in the development of new orthoses. Most of the studies referred to lower extremity devices including ankle-foot orthoses, and focused on a comparison with the traditional methods [23]. Interestingly, a few 3D-printed upper extremity orthoses have been developed. Abdallah et al. developed a 3D-printed myoelectric hand exoskeleton for stroke patients that can aid finger movements. In the study, they proved that the designed orthosis had a positive effect on finger range of motion. They suggested that the system could be considered as a continuous passive motion device [24]. Another study focused on a 3D-printed wrist driven orthosis for patients with SCI. The study showed some improvements in hand function and highlighted the potential possibility of using 3D printing on hand orthosis for patients with SCI. Although the study focused on material properties and the durability of the 3D-printed device, the product did not differ from traditional metal wrist hand orthosis in terms of design, function, and indication [25].

As technology develops, the desire for new hand orthoses is growing. In this study, we proposed a novel myoelectric hand orthosis using 3D printing that provides functional grip for patients with cervical SCI using the tenodesis principle. By using 3D printing technology, we could manufacture a low-cost orthosis suitable for each individual while maintaining essential functionality. Furthermore, we used electromyography (EMG) signals as the input to the controller. The orthosis was designed to be operated under a customized EMG setting and, therefore, could detect the users’ intentions and be controlled more intuitively. The aim of the study was to demonstrate the effects of the 3D-printed myoelectric orthosis in patients with cervical SCI. Accordingly, it was applied to people with chronic cervical SCI and its effects on hand function and daily living were evaluated using clinical tests.

## Methods

### Mechanical design

A computer-aided design software (SolidWorks®, Dassault Systèmes, France) was used to model the 3D architecture of the orthosis. Each individual part was printed with a polylactic acid (PLA) filament, which is easy to use and biodegradable. We used a fused deposition modeling (FDM)-based 3D printer (Moment2®, Moment Co., Ltd., Seoul, Korea). FDM is one of the most widely used manufacturing techniques in 3D printing and has advantages of being able to print products fast and at a low cost [20].

**Fig. 1:**
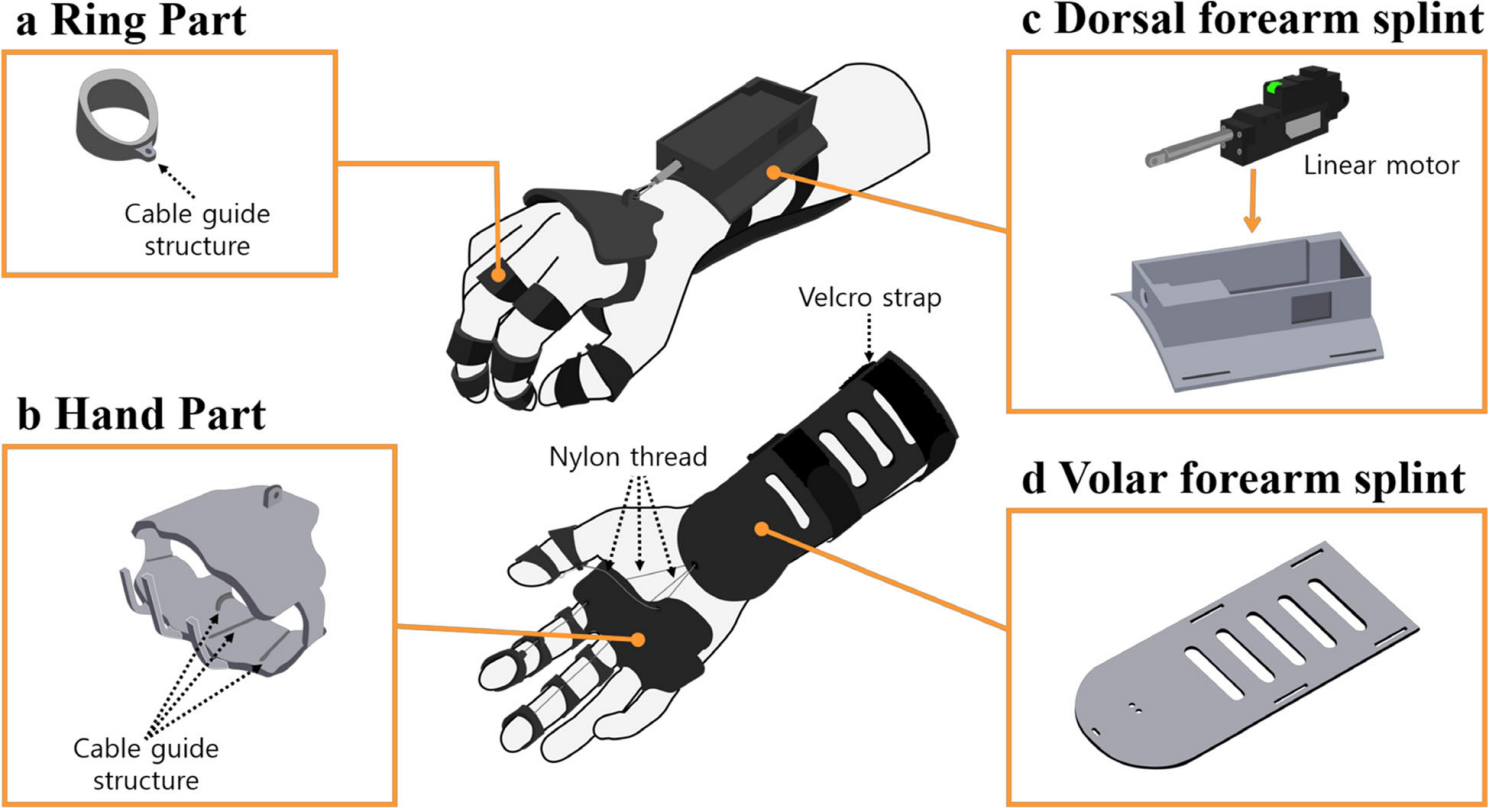
Schematic design of the hand orthosis. **a** Ring part. A total of 8 ring parts were printed for each phalange of the thumb, index, and middle finger. **b** Hand part. **c** Dorsal forearm splint. **d** Volar forearm splint. Note that cable guide structures were designed to the volar side of each finger ring part and hand part to guide the nylon thread

**Fig. 2:**
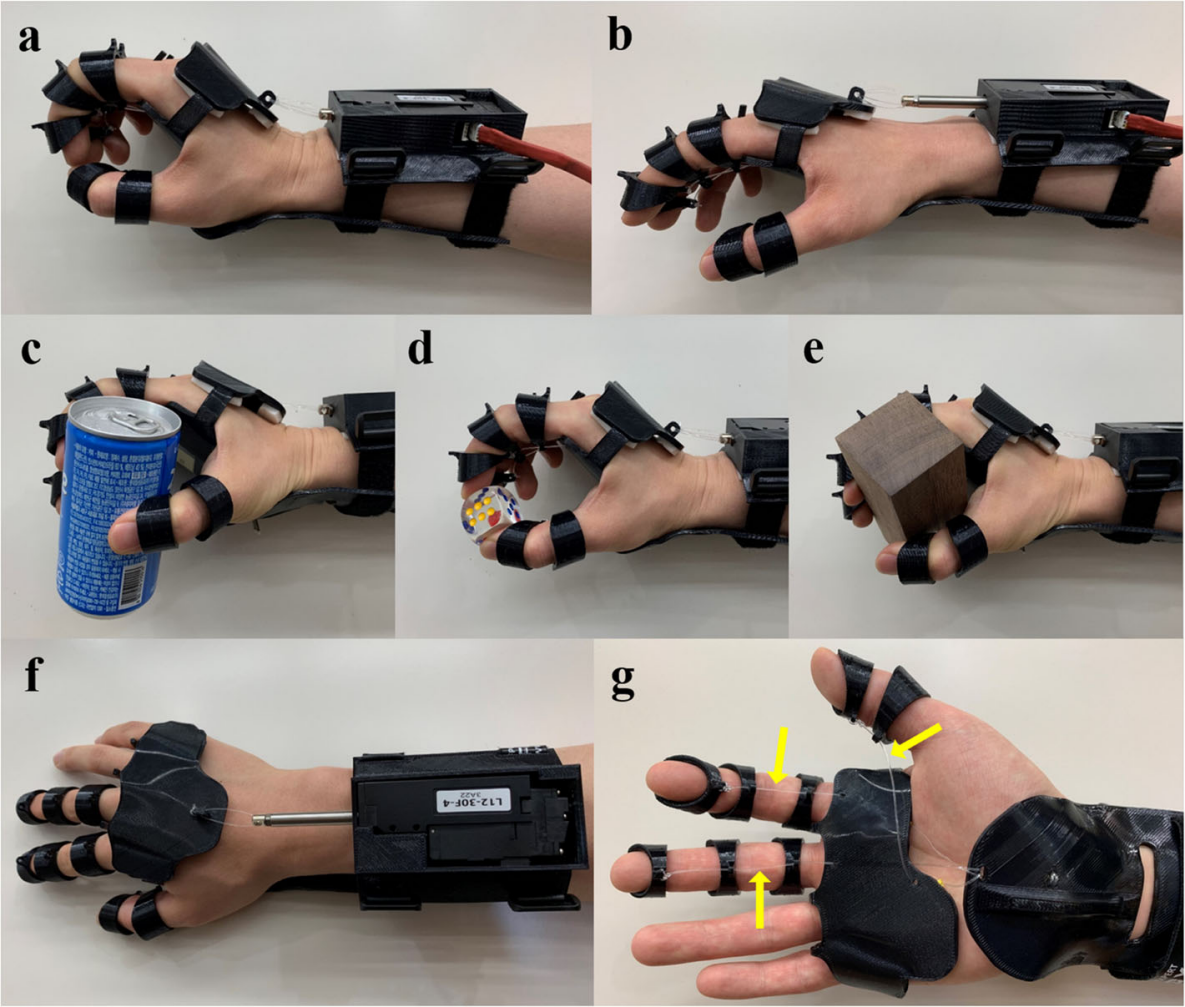
Each motion of the 3D-printed orthosis. **a** When the linear motor is activated by sEMG signals, the wrist is extended, which causes the tenodesis grip. Also, the nylon thread connected to the tip of each finger ring part is tightened as the wrist extended, which enhances grip strength. **b** When the motor goes back into the place, the extended wrist and tension of the nylon thread are released, and the hand becomes in a neutral position. **c-e** A subject is using the orthosis to pick up a pop can, dice, and wooden block, which were used in TRI-HFT. **f** Top view of the orthosis. **g** Bottom view of the orthosis. The arrows indicate nylon thread that connects each finger ring and volar forearm splint

The designed orthosis consists of 3 parts: forearm cuff, hand, and finger ring parts (Fig. 1). The forearm cuff consists of 2 subparts, namely, a dorsal and a volar forearm splint. On the dorsal forearm splint, a linear motor (L12-30F-4®, IR Robot Co., Ltd., Korea), which can generate 30 Newton force with 41 mm stroke length, is mounted to control the wrist extension. The volar forearm splint stabilizes the wrist joint and is bound to the dorsal forearm splint with a velcro strap so that it can be adjusted to the participant’s forearm. The hand part wraps around the hand and anchored to the linear motor. Therefore, when the motor is activated, the wrist part is pulled toward the forearm, which makes the wrist extended. The finger ring parts are located in each phalange of the thumb, index and middle finger, and each volar side of finger ring is connected to the volar forearm splint by nylon thread. Cable guide structures were added to the volar side of each finger ring and hand part to route the nylon thread along the fingertip to the volar forearm splint. When the wrist is extended by the linear motor, the nylon thread tightens, which strengthens the tenodesis grip (Fig. 2). Thus, wrist extension leads to the simultaneous flexion of the interphalangeal and metacarpophalangeal joints of each finger including thumb. Therefore, when an object is placed in the user’s palm or between the fingers, the user can grasp the object by activating the linear motor. This is the key mechanism of orthosis that allows more people to use the device, including patients with high-level SCI who cannot control their wrists. The length of the nylon thread was adjusted so that the finger ring parts could be pulled sufficiently when the wrist is extended.

The handbreadth and circumference of each phalange were measured to make customized orthosis before printing the hand and finger ring parts. The handbreadth was defined as the distance across the palm of the hand at the metacarpal-phalangeal joints of the index to little finger [26]. Because the PLA filament is thermoplastic, all parts printed with the 3D printer were heated using torch gas and re-adjusted to the individual hand shape in detail. After re-adjustment, the self-adhesive pad was attached to the inside of the hand to prevent skin erosion. All mechanical joints were omitted in the orthosis and it was designed to use the patient’s own joint. The wrists of some patients with SCI are radially deviated by radial extensor innervated by C6, which interferes with the effectiveness of the conventional orthosis. By removing the artificial joints of the orthosis, the device can accommodate such a wrist deviation.

The total estimated cost of the developed orthosis is roughly $230 US (Table 1). This includes the price of all components needed to fabricate the orthosis, with the exception of the 3D printer. However, considering that the amount of filament required for each orthosis is estimated to be between 90 and 170 g, depending on its size, and only three surface electromyography (sEMG) electrodes are needed, thus the estimated price is expected to decrease further.

**Table 1:** Cost estimate for the developed orthosis

Components	Estimated cost
PLA filament (1000 g)	$32.50
Linear motor (L12-30F-4®)	$152.50
Arduino (Arduino Mega 2560®)	$35.00
12 V lithium-ion battery	$3.00
Velcro strap	$1.50
Nylon thread	$1.00
sEMG electrode (50EA)	$5.00
Self-adhesive pad	$2.50

### Electronics of the orthosis

The developed myoelectric orthosis was invented to be operated by sEMG recorded in the user’s upper extremity muscles. The control unit was designed to operate the linear motor when the sEMG signal exceeded the preset threshold. To acquire accurate signals, the location of the sEMG electrodes was optimized according to the level of injury and subject’s convenience. We tried to determine appropriate muscles that were easily accessible and preserved after the SCI. In this experiment, either the ipsilateral biceps or the upper trapezius muscle was selected as the target, as all subjects were able to contract the muscles without difficulty. As a result, a pair of sEMG electrodes was located at either the ipsilateral biceps or the upper trapezius muscle depending on the convenience of the subjects. According to the guidelines of sEMG for the non-invasive assessment of muscles, a pair of electrodes was located on the most prominent bulge of the muscle belly and the inter-electrode distance was set as 2 cm [27]. A ground electrode was located at the olecranon of the dominant arm.

**Fig. 3:**
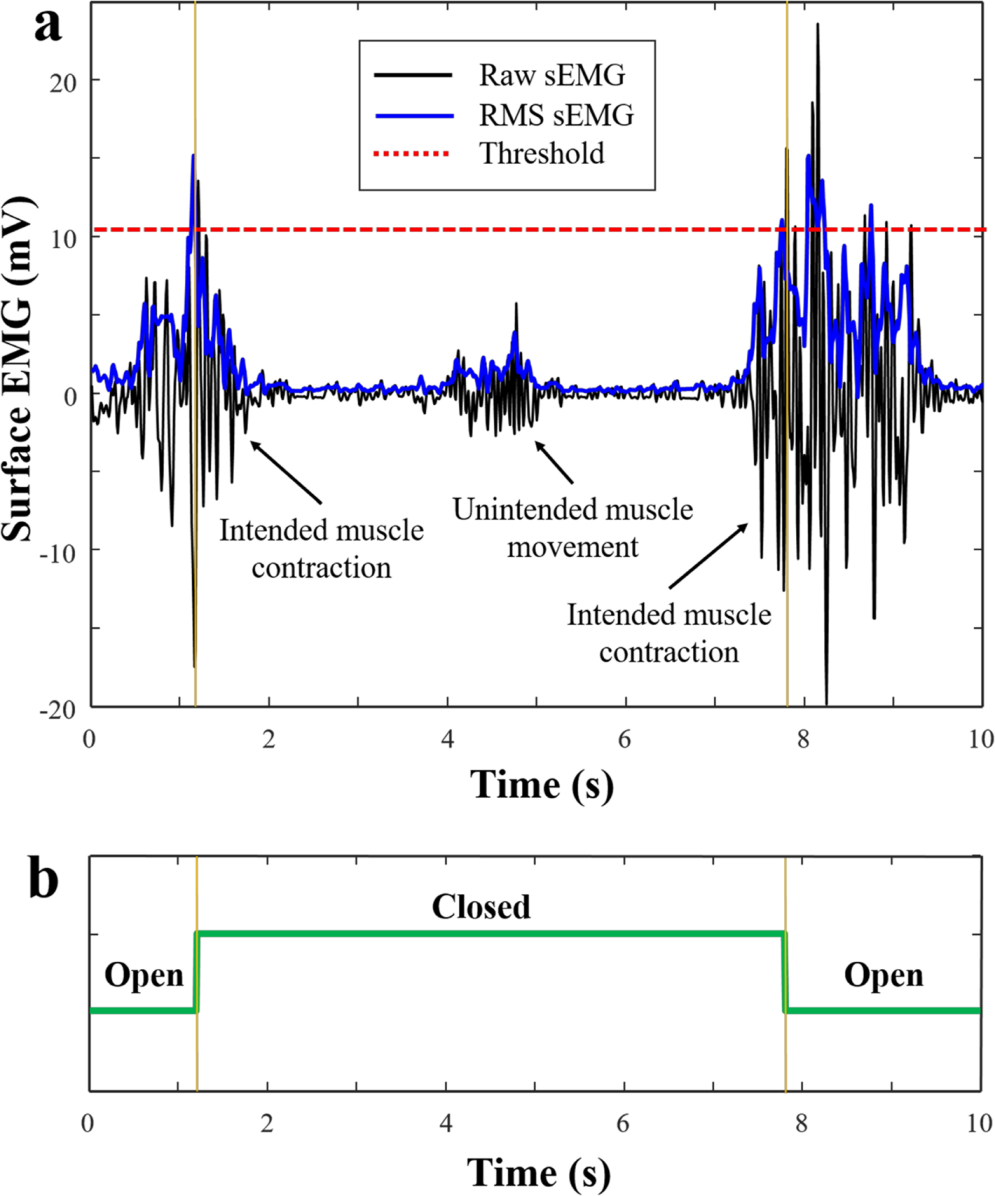
Control mode change of the device according to sEMG signal. **a** Raw and RMS sEMG signal in each situation. Note that the sEMG of usual muscle movements, such as picking up objects and bringing them to oneself, rarely exceeds the preset value (for example, 80% of the maximal RMS value). Therefore, the orthosis was not activated in such contraction level. **b** When the RMS value of the sEMG signal exceeds the threshold value, the myoelectric orthosis activates and the hand is closed by the exoskeleton. If the signal exceeds the threshold again, then the orthosis turns back and the hand opens

Before putting the orthosis on, the sEMG signal was processed. To maximize the quality of the signal, 1000-fold signal amplification was undertaken, and we removed background noise by applying a Sallen-Key band pass filter with 10–500 Hz. The root mean square (RMS) was selected as the parameter for controlling the linear motor because it is one of the most commonly used values in the analysis of EMG signals and is closely related to constant force and muscle contraction [28–30]. Figure 3 shows the raw sEMG signal and RMS for the sEMG signal in each situation. The on/off threshold was set to 80% of the maximal contraction level in RMS in order to distinguish signals from intended and unintended movements. However, the subjects were allowed to adjust the threshold level until they felt comfortable. By doing this, the threshold could be customized according to the users’ ability and injury status. The definition of the RMS can be expressed as follows.

$$ \mathrm{RMS}=\sqrt{\frac{1}{N}\sum \limits_{i=1}^N{X_i}^2} $$

**Fig. 4:**
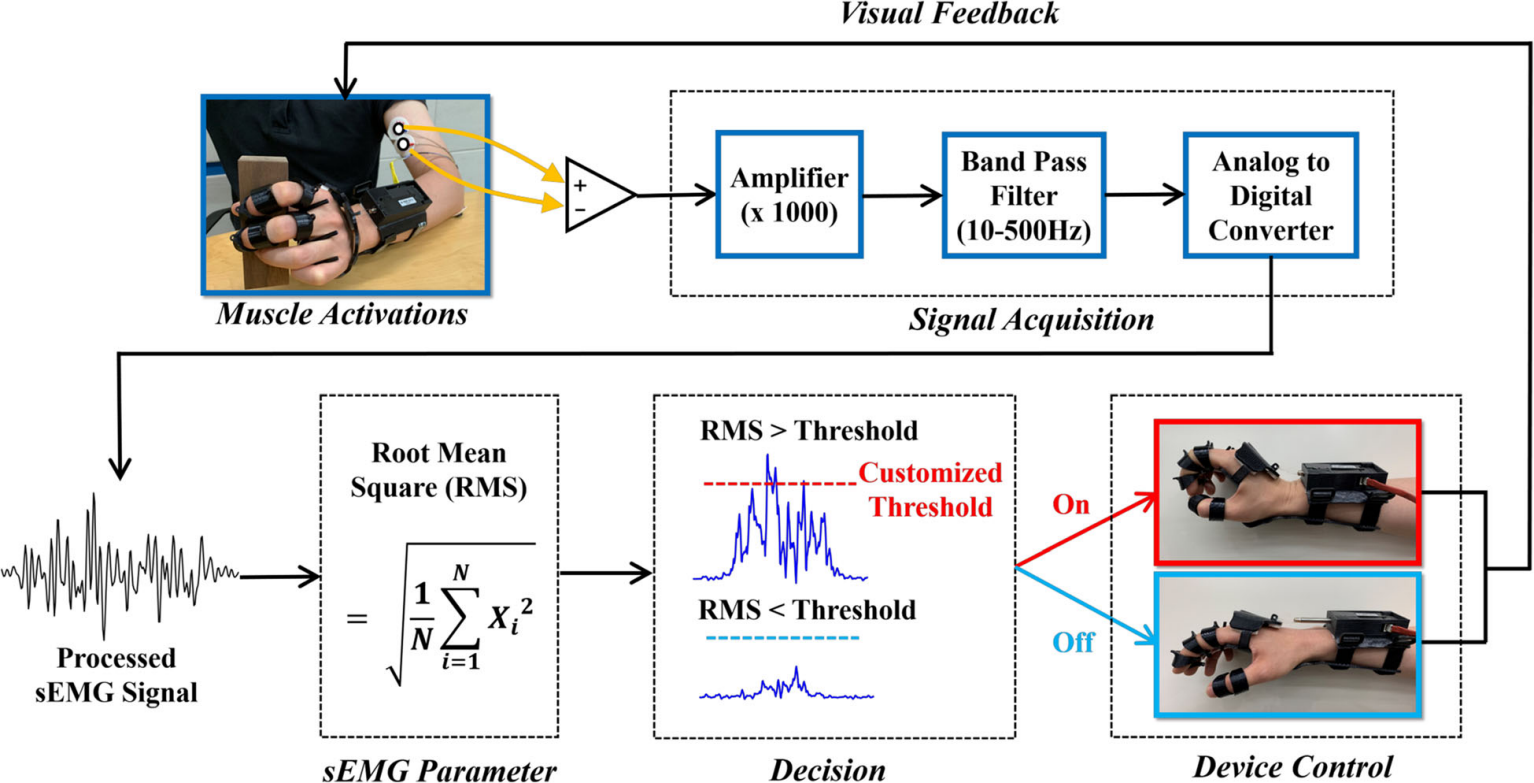
Overview of the control scheme. sEMG signals recorded from the surface electrodes were processed through some acquisition steps such as amplification and band pass filtering in order to improve the signal quality. Then, RMS values of the processed sEMG signals were compared with the customized threshold. The Arduino board classified whether to operate the orthosis according to the magnitude of the RMS value

Arduino (Arduino Mega 2560®, Arduino, Torino, Italy) was used as a microcontroller board. It has an analog input with 10 bits of resolution, and the sampling frequency was 1000 Hz. A 12 V rechargeable lithium-ion battery (F2600®, FAIRMAN Co., Kyonggi do, Korea) was used as a power source for the control unit and the linear motor. The control unit was designed to be as small as possible to maximize the portability. The size and weight of the control box were 10 cm × 5.3 cm × 1.7 cm and 81 g, respectively. The schematic of the control strategy is illustrated in Fig. 4.

### Participants

A total of 10 participants with chronic cervical SCI (9 men, 1 woman; age range, 31–65 years) with stable disability were enrolled in the study (Table 2). They were recruited through the Korea Spinal Cord Injury Association from March 27th, 2019 to April 31st, 2019 on the basis of the following inclusion criteria: (1) age between 18 and 65 years, (2) at least 12 months after cervical SCI, (3) neurological level of injury from cervical 4 to 7 (C4 to C7) according to American Spinal Injury Association (ASIA) guideline, and (4) impairment of hand function due to SCI. Those who met 1 of the following criteria were excluded from the study: (1) a medical history of other coexisting neurological injuries (e.g, traumatic brain injury, stroke, or cerebral palsy), (2) severe hand deformity or spasticity (Modified Ashworth scale (MAS) ≥ 3) [32], (3) severe neuropathic pain on the upper extremity after the SCI, (4) severe orthostatic hypotension, (5) having unstable spinal fractures, (6) persistent other medical conditions (e.g, cardiopulmonary disease, or infection), or (7) pregnant women.

**Table 2:** Clinical characteristics of the study participants

Participants	Age	Gender	ASIA Impairment Scale	NLI	Years Since Injury	Finger Flexor Motor grade^a^	Finger Flexor Spasticity (MAS)
1	43	Female	ASIA D	C4	17 years	1	1
2	36	Male	ASIA A	C5	14 years	1	0
3	65	Male	ASIA A	C4	28 years	0	0
4	52	Male	ASIA A	C7	26 years	2	0
5	51	Male	ASIA A	C6	15 years	2	1
6	63	Male	ASIA C	C6	5 years	3	1+
7	56	Male	ASIA C	C5	28 years	1	1
8	31	Male	ASIA B	C6	5 years	2	0
9	51	Male	ASIA B	C6	17 years	1	1
10	53	Male	ASIA C	C7	23 years	0	0

*ASIA* American Spinal Injury Association, *NLI* Neurological Level of Injury, *MAS* Modified Ashworth scale

^a^The motor grade was evaluated using manual muscle testing developed by the Medical Research Council [31]

Prior to the study, participants were instructed regarding the experiment, and all provided written informed consent. The study was approved by the Clinical Research Information Service (KCT0003995) and Institutional Review Board of the Gwangju Institute of Science and Technology (20190327-HR-43-01-02).

### Experiment procedure

Before the experiment, all the participants underwent thorough clinical examination by a physiatrist with many years of experience in this field. The motor and sensory function of the upper extremities along with the neurologic level of injury, according to the ASIA guidelines, were evaluated. Furthermore, spasticity of the dominant hand was evaluated using MAS.

The experiment was designed to compare the participant’s situation with and without the orthosis. Prior to donning the orthosis, participants underwent baseline measurements of hand function and functional independence in daily living. Baseline measurement without the orthosis might create a bias to the evaluation because it is not blinded. Accordingly, evaluating baseline conditions while wearing the orthosis with the motor being passive might be a better method. However, we decided to evaluate the baseline condition with their bare hands because it reflects the actual baseline hand function. Hand function of the dominant side was evaluated using the Toronto Rehabilitation Institute Hand Function Test (TRI-HFT) for the primary outcome measure [33]. This simple test was specially designed to evaluate the unilateral gross motor function of patients with C4 to C7 SCI using standardized objects that might be encountered in daily living. The TRI-HFT is known to be sensitive enough to assess the change in hand function under different conditions in patients with SCI. Therefore, many previous studies have used this assessment tool to prove the effectiveness of hand assistive devices or therapeutic interventions [33–37]. The test consists of 2 parts: the first part of the test evaluates the ability to manipulate objects with 10 standardized items, and the second part evaluates the strength of the grasp using 9 rectangular wooden blocks, an instrumented cylinder and credit card attached to a dynamometer, and wooden bar. The 9 wooden blocks of different weights and frictions are used to evaluate the strength and stability of grasp, while the three other items are used to measure the torque generated by palmar grasp, lateral pinch force, and eccentric load that the grasp could sustain, respectively. However, the latter tree items have not been validated yet and primarily focus on evaluating forces exerted by the motorized hand exoskeleton [33, 37]. Therefore, we evaluated the hand function using 10 items in the first part and 9 wooden blocks in the second part of the test. Each subset is scored on a scale of 0 to 7 based on the TRI-HFT criteria; therefore, the total maximum scores for the 2 parts was 133 points. There was no time limit in performing the task. Participants were allowed to terminate each task when they thought they had accomplished the task to the best of their capabilities. Furthermore, sufficient rest was allowed to minimize muscle fatigue. The whole assessment was recorded on video, and 2 independent observers scored the test according to the TRI-HFT guidelines to minimize observer bias. Sufficient discussion to achieve a consensus occurred between the observers if there were any difference in test scores.

For the secondary outcome, functional independence in daily living was evaluated using the Functional Independence Measure (FIM) self-care subscale, and the Spinal Cord Independence Measure (SCIM) III self-care subscale. The evaluation was undertaken by observation and interview. The FIM is the most widely used functional independence measurement tool and has been used in many rehabilitation communities. It assesses functional ability in 6 distinct areas (self-care, sphincter management, transfers, locomotion, communication, and social cognition) [38, 39]. Among them, we evaluated the FIM self-care subscale only (6 items, subscores 0–42) as this subscale is most likely to be affected by hand function. SCIM III, the second functional independence measurement tool, is the latest version of SCIM that is developed specifically for patients with SCI. It evaluates the ability of performance in activities of daily living (ADL) and is known to be the most sensitive, reliable, and valid measurement for individuals with SCI [40, 41]. The SCIM consists of 3 subscales, namely, self-care, respiration and sphincter management, and mobility. We only evaluated the self-care subscale (4 items, subscores 0–20), which is relevant to the hand function. Finally, the Korean-Quebec User Evaluation of Satisfaction with Assistive Technology 2.0 (K-QUEST 2.0) was used to evaluate a person’s satisfaction with several components of the orthosis [42, 43]. K-QUEST 2.0 is a 12-item outcome measurement tool rated on a 5-point satisfaction scale (range 1 to 5). It assesses user satisfaction in 2 aspects, device and services. We only surveyed with regard to device dimension, which consisted of 8 items.

After the baseline measurements, participants put the myoelectric orthosis on their dominant hand, and improvements in hand function and functional independence were evaluated using the same methods. After finishing the experiment, a careful observation was done to see if there were any side effects related to the experiment, including pressure ulcers. Since the participants had limitations with regard to independent walking due to SCI, the experiments were conducted at the Gwangju Spinal Cord Injury Association Center or the participants’ home, depending on what was more convenient for the participants.

### Statistical analysis

All functional measurements, including TRI-HFT, FIM, and SCIM III, were evaluated in all participant. Non-parametric statistics were used as the sample size was small, and data did not show normal distribution in the Shapiro–Wilk test (*p*-value < 0.05). Therefore, the Wilcoxson signed-rank test was used to assess the improvement of total scores of functional measurements before and after wearing the orthosis. Additionally, the false discovery rate method was applied to control the multiple testing problem. All statistical analyses were conducted using MATLAB version 2017b (Mathworks, Inc.). For all tests, statistical significance was set at 0.05.

## Results

### Improvements of the hand function with the orthosis

After completing the experiment, the performance of the orthosis was analyzed by comparing hand function before and after wearing the orthosis. The results of the TRI-HFT are summarized in Table 3. Additionally, the TRI-HFT scores of each participant are summarized in Additional file 1. Although the myoelectric orthosis provided efficient grip by enhancing the tenodesis, it was not designed to offer individual finger movements. Therefore, it was difficult for subjects to manipulate objects using active finger grasp. As a result, the highest score that can be achieved on the TRI-HFT for each item was 6 out of 7 points, which stands for lifting the object completely off and manipulating them using passive grasp, such as a tenodesis grip. The first part of the TRI-HFT evaluates grasp, lift, and object manipulation. In the first part, the total average score was improved from 29.40 to 45.00 points. Furthermore, the improvement was statistically significant on the Wilcoxson signed-rank test (*p* = 0.007). When analyzing each item, the orthosis was proven to be effective in dealing with the majority of objects. However, there were no significant improvements in score when dealing with small or relatively flat objects such as a book, credit card, mobile phone, or pencil. With regard to the second part of the test, which estimates strength and stability of grip, participants could hold and move wooden blocks efficiently. Regardless of the weight and friction of the blocks, subjects could handle the objects while using the orthosis. Exceptionally, 1 participant (subject 7) performed worse with the orthosis than without the orthosis (Additional file 1). The participant had suffered from cervical cord injury for 28 years and had some degree of flexor contracture on his fingers and wrist. Furthermore, he was accustomed to grasping objects with his contracted hand for a long time. As a result, he felt uncomfortable with the new grip pattern provided by the developed orthosis, and the effect of the device was reduced due to hand deformity. Other than that, most subjects were able to acquire scores close to 6 points on every block and showed statistically significant improvements (*p* < 0.05). Although the statistically significant improvements might not imply that they are actually meaningful, most participants could grasp and lift the objects after wearing the orthosis, which were impossible at the baseline measurements. From this, we could infer that the improvements of TRI-HFT score are clinically meaningful.

**Table 3:** TRI-HFT of the study participants at before and after wearing the orthosis

	Score		*p*-value
	Base-line	Assisted with orthosis	
First Part: Object Manipulation			
1. Mug	3.5	5.6	**0.035**
2. Paper	3.2	5.2	**0.047**
3. Book	2.3	3.8	0.126
4. Ziploc bag	3.2	4.6	**0.047**
5. Pop can	2.8	4.6	**0.047**
6. Dice	2.9	5.6	**0.035**
7. Sponge	2.4	4.8	**0.047**
8. Credit card	3.1	3.6	0.574
9. Mobile phone	2.9	3.2	0.522
10. Pencil	3.1	4.0	0.246
Total score for the First Part	29.4	45.0	**0.007**
Second Part: Rectangular Wooden Blocks			
100 g block; high friction surface	3.4	5.6	**0.012**
100 g block; wooden surface	3.0	5.6	**0.012**
100 g block; low friction surface	3.0	5.4	**0.012**
200 g block; high friction surface	3.2	5.4	**0.012**
200 g block; wooden surface	2.8	5.2	**0.012**
200 g block; low friction surface	2.8	5.2	**0.012**
300 g block; high friction surface	3.2	5.2	**0.012**
300 g block; wooden surface	2.6	5.2	**0.012**
300 g block; low friction surface	2.8	4.8	**0.012**
Total score for the Second Part	26.8	47.6	**0.009**

Bold values indicate statistically significant differences. False discovery rate method was applied to control the multiple testing problem. Besides, the Wilcoxon signed-rank test was used to analyze the improvement of the total TRI-HFT scores after wearing the orthosis

### Improvements of the ADL with the orthosis

ADL was evaluated using FIM and SCIM III before and after the experiment, and the improvement was analyzed (Table 4). The detailed improvement of each subject is reported in Additional file 2. In general, the evaluation of ADL is appraised at sufficient time intervals. For example, FIM is usually evaluated within 72 h of admission to a rehabilitation facility and reevaluated within 72 h before discharge [38, 39]. However, in order to assess the immediate improvement of ADL without sufficient adaptation, SCIM III and FIM were remeasured on the same day that the orthosis was applied. After wearing the orthosis, significant improvements were observed in the total SCIM III and FIM scores. However, when analyzing ADL by each task, it showed improvements only in the eating category of the FIM score, the category that was the most affected by hand function. On the other hand, there was no significant improvement in other tasks, such as grooming, which requires fine motor function, or bathing and closing, which also need trunk balance or lower extremity function.

**Table 4:** ADL of the study participants at base-line and with orthosis

Task	Base-line	Assisted with orthosis	*p*-value
Functional Independent Measurement (FIM): Self-care			
Eating	3.5	5.4	**0.035**
Grooming	1.9	2.1	0.549
Bathing	1.5	1.5	1.0
Dressing-Upper Body	2.0	2.0	1.0
Dressing-Lower Body	1.1	1.1	1.0
Toileting	1.3	1.3	1.0
Total Score	11.3	13.4	**0.0004**
Spinal Cord Independence Measure (SCIM) III: Self-care			
Eating	2.2	3.4	0.104
Bathing	0.6	0.9	0.166
Dressing	0.9	0.9	1.0
Grooming	0.9	0.9	1.0
Total Score	4.6	6.1	**0.027**

Bold values indicate statistically significant differences. False discovery rate method was applied to control the multiple testing problem. Besides, the Wilcoxon signed-rank test was used to analyze the improvement of the total ADL scores after wearing the orthosis

### Feedback from the subjects

After completing the TRI-HFT with the myoelectric orthosis, subjects were asked to rate the orthosis using K-QUEST 2.0. They evaluated the orthosis across 8 items; dimensions, weight, adjustments, safety, durability, simplicity of use, comfort, and effectiveness, and scored each item on the scale from 1 (not satisfied at all) to 5 (very satisfied) (Table 5). Most subjects were satisfied with the effectiveness of the orthosis. The average item score regarding effectiveness was around 4.5 and it was the highest ranked across the 8 items. However, some participants voiced that the dimension of the orthosis was a little bit bulky due to the linear motor and was hard to adjust by themselves. Otherwise, the participants were satisfied overall with the developed orthosis. Furthermore, none of the participants requested additional time to adjust the system since the control scheme based on sEMG was easy to understand. None of the subjects complained about any discomfort related to the study, and no side effects including pressure ulcer were found during the experiment.

**Table 5:** Results of the survey using K-QUEST 2.0; device domain

	Items	Satisfaction
Assistive Device Domain	Dimensions	3.2
	Weight	3.8
	Adjustments	3.4
	Safety	4.1
	Durability	3.8
	Simplicity of use	3.9
	Comfort	3.8
	Effectiveness	4.5

## Discussion

In the present study, we proposed a novel hand orthosis using 3D printing techniques that enhanced tenodesis grip and successfully assisted users’ ability to perform hand function in everyday life. Furthermore, the assisted device received high ratings in the usability test, particularly regarding effectiveness. Participants who could not manipulate objects with their hand showed improvements in hand function and ADL (related to eating) with the help of the orthosis. Other ADL-related subscales, such as self-care or mobility, were not improved because such ADL activities require additional abilities such as lower extremity strength or trunk balance. In the baseline measurements, participants showed poor performance on the hand function test. However, they could securely grasp and lift the objects with their volition after putting the orthosis on. The majority of subjects scored close to 6 points in the second part of the TRI-HFT, regardless of the weight or friction of the wooden blocks. This might be because the blocks were of the proper shape and size to grasp. However, considering that the low-friction wooden block of 300 g could be lifted by the majority of subjects, we could conclude that the orthosis offered sufficient grip strength and the ability of hand function to perform a wide range of daily tasks.

Generally, patients with cervical cord injury who are capable of wrist extension but have impaired hand function, for example, C6 injuries, receive occupational therapy to improve grip using passive grasp called tenodesis. This refers to the natural closing of the fingers into the palm when the wrist is extended [15]. The active wrist extension makes finger flexion as the tendons of the flexor digitorum profundus and flexor digitorum superficialis are stretched. It is a compensatory passive prehension movement, and physiatrists often prescribe a hand splint to shorten the finger flexor muscles and promote the effectiveness of the tenodesis grip [44]. It seems fascinating that a person with no hand function at all can compensate through voluntary movements of the wrist. Nonetheless, this passive grasp is relatively so weak that there are limits to dealing with a variety of things in everyday life [45]. To overcome such a condition, wrist-driven wrist hand orthosis, such as the Rehabilitation Institute of Chicago plastic tenodesis splint, is often prescribed to those patients to enhance the tenodesis grip. Recently, a soft wearable hand robot that uses a soft tendon routing system was developed to assist people with hand disability. Since the soft hand orthosis did not require any of the rigid frames, it was lighter and more compact than the conventional hard orthosis [46]. However, those two orthoses have a limitation in that only patients with functional wrist extension can use the devices. Ratchet-style wrist hand orthosis can be an alternative option for individuals with insufficient wrist extension power. However, this is one of the externally powered orthoses, which has the disadvantage of not being able to control directly by user’s own power [47]. Therefore, we tried to overcome the limitations of previous devices and develop a dynamic hand orthosis based on tenodesis phenomenon, which can be used independently and effectively by more patients.

The dynamic hand orthosis developed in this study has specific characteristics and advantages compared to the other exoskeleton system being developed at the recent stage of research. First, the orthosis has some structural advantages. Above all, it weighed less than most of the newly developed devices because it was manufactured using 3D printing technology with simple control scheme. The total weight of the exoskeleton attached to the hand was < 190 g (85–90 g for the 3D-printed hardware depending on the size and 96 g for the linear motor). Hybrid electroencephalography (EEG) and electrooculography (EOG)-based hand exoskeleton developed for the assistance of hand opening and closing was made of titanium alloy and weighted 438 g [37]. Even current 3D-printed hand exoskeletons for individuals with SCI also weighed > 300 g [48, 49]. Randazzo L et al. tried to develop hand exoskeleton which was as lightweight as possible and the overall weight of the exoskeleton was < 50 g. However, the control box weighed about 930 g, which was designed to be mounted on the user’s chest. Therefore, the overall design was bulky [50]. Various well-designed pneumatic or hydraulic actuators have also been developed [34, 51–53] and have advantages of being lightweight and having low stiffness. However, they also have the potential disadvantage of minimizing the size of the control unit. For instance, a soft robotic glove controlled by pneumatic pump weighed 77 g, but the control box was 5 kg [34]. In contrast, the control box of our device was around 260 g (81 g for the control unit and 182 g for the lithium-ion battery), which made it more portable. Besides, the use of hydraulic pumps requires additional space for water storage, and there is always a risk of fluid leakage [53].

Another structural advantage of the orthosis was that the artificial joints of the device were omitted to make it as simple as possible. As a result, not only was it possible to minimize the weight of the device but by using the user’s joints, it was also possible to grab objects without disturbing the natural finger movement trajectory. Although only tenodesis grasp or three-jaw chuck grasp is possible in the developed orthosis, this type of grasp pattern accounts for almost 80% of all prehension grasps [54]. Therefore, the subjects were able to handle everyday objects and show better performance on the TRI-HFT test and ADL measurement. Besides, the size was easily scalable and can be custom-fitted according to the condition and size of the patient’s hand. By simply measuring the handbreadth and the circumference of the fingers, we could easily produce personalized hand orthosis.

Second, the developed orthosis is based on a myoelectric control system that utilizes sEMG signals. EMG, which represents the electrical activity of contracted muscles, is widely studied and is the most common approach for the control of assistive devices. Although there are some challenges to overcome, such as signal processing or electrode placement, previous studies have indicated that control schemes using sEMG are effective in recognizing the user’s intentions and feasible for controlling power-assist devices as it directly reflects the targeted muscle activity in real time [55–58]. The developed orthosis also utilizes sEMG signals from the user’s arm to control the device. This allowed for more intuitive and voluntary control of the device and increased patient autonomy. In contrast, some of the previously developed wearable hand robotics were less intuitive because they were controlled by another operator other than the subject [34, 53]. A very advanced and lightweight polymer-based soft wearable hand robot was recently developed, and it was activated according to the user’s intention. Although the actuation system was rather large, an intention button was designed so that the users can press it to actuate the device. Push-button operated devices are very simple and can be controlled accurately over extended periods. In particular, the control signals from the push-button operated device are rarely affected by movement artifacts or sweating, which are the major obstacles of the myoelectric control system. However, it is less physiological than the myoelectric devices because it might need the help of another hand to press the button [59]. On the other hand, the orthosis of this study has advantages in terms of control because it can be controlled without the help of another hand because it is operated directly by contraction of the unilateral target muscle. In addition, it was possible to control the device more easily by customizing the sEMG threshold value. By using the simple on/off strategy using the threshold, computational power and hardware complexity could be minimized. Furthermore, the sEMG electrodes could be attached according to the user’s status and no special training time was needed because only a simple contraction of the targeted muscle is required to manipulate the device. Therefore, it could be applied to more people with SCI than conventional orthosis. Specifically, traditional tenodesis splints are used only in patients with functional wrist extension, whereas the developed orthosis can be applied in any participant who can control their proximal upper extremity. Besides, it can also aid the hand function of subjects who are able to control their wrist. Although the subjects 4 and 10 were able to perform some degree of wrist extension, the tenodesis grip was not effective due to the lack of stiffness in their hand. However, these patients also showed improvements in hand function with the help of this orthosis.

Although we developed a low-cost and lightweight 3D-printed hand orthosis using a simple design and control scheme, it showed similar performance to other newly developed assistive devices that were more complicated. For instance, a cable-driven myoelectric orthosis was developed, and its efficacy was evaluated using the Sollerman hand function test. The hand exoskeleton was well-controlled by sEMG signals and could perform four types of hand movements. However, it was bulky, and the hand function test score did not dramatically improve [49]. Cappello et al. developed a new fabric-based soft robotic glove, which was controlled by a pneumatic pump. It was lightweight with compliant design and could control each individual finger. Therefore, it enhanced dexterous movements and showed improvement of hand function on TRI-HFT, which were similar to the results of the present study. However, it needed additional space to locate the control box since it was relatively large [34]. A hybrid EEG and EOG-based hand exoskeleton was very intuitive and showed almost full restoration of hand function in paraplegic individuals. However, the exoskeleton needed a wireless tablet computer and actuators to control the system. Furthermore, it also required more complicated hardware such as a cap to record EEG [37]. The cost-effectiveness ratio can be defined as the net cost to the net improvement in health in the healthcare system [60, 61]. The orthosis was made as simple as possible and utilized 3D printing technology to minimize the net cost. Besides, the 3D printing technology has enabled us to produce customized orthosis in a short time. Although the studies mentioned previously did not provide the total cost for the devices, the orthosis developed in this study seems cost-effective considering that we can manufacture the device promptly and achieve similar effects as others at a low cost.

In the present study, we evaluated user satisfaction with the device using K-QUEST 2.0. Even though the survey of user satisfaction is considered to be important for patient-centered research, few studies have conducted a usability test when developing an assistive device. Another reason to do the usability test is that it allows the researcher to compare the subjectivity of newly developed devices with existing devices [62]. A study evaluated the overall satisfaction of orthoses in Korean people using K-QUEST [63]. QUEST 2.0 is a measurement test that was developed in Canada to evaluate user’s satisfaction with a variety of assistive technology [42]. K-QUEST 2.0 is the Korean version of QUEST 2.0 and shares the same document form of the test [43]. Compared with the results of the previous study, participants scored higher on the items of “Safety,” “Simplicity of use,” “Comfort,” and “Effectiveness” in this experiment. In particular, the item with “Effectiveness” received the highest score, which is about 0.5 points higher than reported in the study we referred to. However, the developed orthosis received lower scores on the items about “Dimensions” and “Adjustments.” Despite the limitations, the majority of patients said the developed orthosis effectively enhanced hand function and was comfortable to wear because it was designed to fit the shape of an individual’s hand using 3D printing.

Because the primary goal of many assistive devices is to improve the functionality of our body, we think that this study has achieved remarkable results. However, we believe that this 3D-printed myoelectric hand orthosis can be further improved in the future. First, it is imperative to improve the dimensions and adjustments of the device based on feedback from the users. The size of the linear motor needs to be reduced, or other types of motors, such as a direct current motor or servo motor can be an alternative. Second, printing the device with flexible filaments such as dielectric elastomers or hydrogels might improve adjustments and comfort [64]. Furthermore, strengthening the grip force by improving the power of the mounted motor or attaching additional structures to increase friction between the fingers and objects will expand the usability of the device because the current device does not support large grasp forces. The control scheme can be advanced to closed-loop systems to improve the task performance and usability as bi-directional communication between the exoskeleton and user is one of the ultimate goals of human-machine interface [65]. Embedding proprioceptive sensors, such as pressure or resistive bend sensor, in the exoskeleton can be a good clue for the development of a closed-loop system [66]. Finally, training the intended muscle contraction and unintended muscle movements using a machine learning technique will optimize the personalized threshold value for device activation. Further study is needed to improve the structural components and robustness of the control system over extended periods of time.

## Conclusions

In order to help more physically challenged people, assistive devices based on low-cost and simple technology are pivotal in the rehabilitation field. In this study, we presented a newly developed myoelectric hand orthosis using 3D printing. The dynamic orthosis significantly improved the hand function and ADL of people with cervical SCI in clinical tests including the TRI-HFT. It also received positive feedback in the usability test. Although we have not evaluated long-term usability, it is expected that users will benefit more from the orthosis if they have sufficient training time to adapt to the system. Furthermore, if we optimize the orthosis in terms of volume and adjustments, it can be used in the clinic soon and extended to other neurologically injured people, such as stroke or brachial plexus injury patients. It is still an early stage of research, and some areas need to be improved. However, this 3D-printed myoelectric hand orthosis seems to be cost-effective and promising to utilize as an alternative to conventional devices. We hope that this study will be the cornerstone of research on assistive devices using 3D printing technology.

## Supplementary information

**Additional file 1.** Distribution of the TRI-HFT scores across each individual. a Results of the TRI-HFT score in the first part. Most subjects showed improvements in hand function after wearing the orthosis but showed mixed results when dealing with small or flat objects such as a book, credit card, mobile phone, or pencil. b Results of the second part of the TRI-HFT score. The majority of subjects showed improvements in hand function with the help of the orthosis.

**Additional file 2.** Distribution of ADL scores across each individual. Functional improvements were observed in the eating category. However, few improvements or no changes were observed in the remaining categories. **a** Results of the FIM self-care subscale. **b** Results of the SCIM III self-care subscale.

## Data Availability

Please contact the authors for data requests.

## Abbreviations

3D: three-dimensional
ADL: Activities of daily living
ASIA: American spinal injury association
CAD: Computer-aided design
EEG: Electroencephalography
EMG: Electromyography
EOG: Electrooculography
FDM: Fused deposition modeling
FIM: Functional independence measure
K-QUEST: Korean-Quebec user evaluation of satisfaction with assistive technology
MAS: Modified Ashworth scale
PLA: Polylactic acid
RMS: Root mean square
SCI: Spinal cord injury
SCIM: Spinal cord independence measure
sEMG: surface electromyography
TRI-HFT: Toronto rehabilitation institute hand function test

## References

[1] Badhiwala JH, Wilson JR, Fehlings MG (2019). Global burden of traumatic brain and spinal cord injury. Lancet Neurol.

[2] Cripps R, Lee B, Wing P, Weerts E, Mackay J, Brown D (2011). A global map for traumatic spinal cord injury epidemiology: towards a living data repository for injury prevention. Spinal Cord.

[3] Ning G-Z, Wu Q, Li Y-L, Feng S-Q (2012). Epidemiology of traumatic spinal cord injury in Asia: a systematic review. J Spinal Cord Med.

[4] Jazayeri SB, Beygi S, Shokraneh F, Hagen EM, Rahimi-Movaghar V (2015). Incidence of traumatic spinal cord injury worldwide: a systematic review. Eur Spine J.

[5] Krassioukov A (2009). Autonomic function following cervical spinal cord injury. Respir Physiol Neurobiol.

[6] Winslow C, Rozovsky J (2003). Effect of spinal cord injury on the respiratory system. Am J Phys Med Rehabil.

[7] Snoek GJ, IJzerman MJ, Hermens HJ, Maxwell D, Biering-Sorensen F (2004). Survey of the needs of patients with spinal cord injury: impact and priority for improvement in hand function in tetraplegics. Spinal Cord.

[8] Ragnarsson K (2008). Functional electrical stimulation after spinal cord injury: current use, therapeutic effects and future directions. Spinal Cord.

[9] Nas K, Yazmalar L, Şah V, Aydın A, Öneş K (2015). Rehabilitation of spinal cord injuries. World J Orthop.

[10] Burns AS, Ditunno JF (2001). Establishing prognosis and maximizing functional outcomes after spinal cord injury: a review of current and future directions in rehabilitation management. Spine.

[11] Bertelli JA, Ghizoni MF (2017). Nerve transfers for restoration of finger flexion in patients with tetraplegia. J Neurosurg Spine.

[12] Ward JA, Power DM (2019). Nerve transfers following cervical spinal cord injury: a review and reconstructive algorithm. J Musculoskelet Surg Res.

[13] Vanmulken D, Spooren A, Bongers H, Seelen H (2015). Robot-assisted task-oriented upper extremity skill training in cervical spinal cord injury: a feasibility study. Spinal Cord.

[14] Curt A, Hsieh J, Schubert M, Hupp M, Friedl S, Freund P (2019). Safety and preliminary efficacy of allogeneic neural stem cell transplantation in chronic spinal cord injury: a translational phase I/IIa trial.

[15] Cifu DX (2016). Braddom’s Physcial Medicine and Rehabilitation.

[16] Sutton S (1993). An overview of the management of the C6 quadriplegic patient's hand: an occupational therapist's perspective. Br J Occup Ther.

[17] PowerGrip Assisted Grasp Orthosis. https://inclusivetechnologies.org/products/powergrip. Accessed 3 May 2019.

[18] Schubert C, Van Langeveld MC, Donoso LA (2014). Innovations in 3D printing: a 3D overview from optics to organs. Br J Ophthalmol.

[19] Popov VV, Muller-Kamskii G, Kovalevsky A, Dzhenzhera G, Strokin E, Kolomiets A (2018). Design and 3D-printing of titanium bone implants: brief review of approach and clinical cases. Biomed Eng Lett.

[20] Gross BC, Erkal JL, Lockwood SY, Chen C, Spence DM (2014). Evaluation of 3D printing and its potential impact on biotechnology and the chemical sciences. Anal Chem.

[21] Mamidi SK, Klutcharch K, Rao S, Souza JC, Mercuri LG, Mathew MT (2019). Advancements in temporomandibular joint total joint replacements (TMJR). Biomed Eng Lett.

[22] Ventola CL (2014). Medical applications for 3D printing: current and projected uses. Pharm Ther.

[23] Lunsford C, Grindle G, Salatin B, Dicianno BE (2016). Innovations with 3-dimensional printing in physical medicine and rehabilitation: a review of the literature. PM R.

[24] Abdallah Ismail Ben, Bouteraa Yassine, Rekik Chokri (2017). DESIGN AND DEVELOPMENT OF 3D PRINTED MYOELECTRIC ROBOTIC EXOSKELETON FOR HAND REHABILITATION. International Journal on Smart Sensing and Intelligent Systems.

[25] Portnova AA, Mukherjee G, Peters KM, Yamane A, Steele KM (2018). Design of a 3D-printed, open-source wrist-driven orthosis for individuals with spinal cord injury. PLoS One.

[26] Pheasant S (2014). Bodyspace: anthropometry, ergonomics and the design of work: anthropometry, ergonomics and the design of work: CRC press.

[27] Hermens Hermie J, Freriks Bart, Disselhorst-Klug Catherine, Rau Günter (2000). Development of recommendations for SEMG sensors and sensor placement procedures. Journal of Electromyography and Kinesiology.

[28] Boostani R, Moradi MH (2003). Evaluation of the forearm EMG signal features for the control of a prosthetic hand. Physiol Meas.

[29] Kim KS, Choi HH, Moon CS, Mun CW (2011). Comparison of k-nearest neighbor, quadratic discriminant and linear discriminant analysis in classification of electromyogram signals based on the wrist-motion directions. Curr Appl Phys.

[30] Phinyomark A, Phukpattaranont P, Limsakul C (2012). Feature reduction and selection for EMG signal classification. Expert Syst Appl.

[31] Riddoch G, Committee MRCNI (1943). Aids to the investigation of peripheral nerve injuries: HM stationery office.

[32] Charalambous Charalambos P. (2013). Interrater Reliability of a Modified Ashworth Scale of Muscle Spasticity. Classic Papers in Orthopaedics.

[33] Kapadia N, Zivanovic V, Verrier M, Popovic M (2012). Toronto Rehabilitation Institute–hand function test: assessment of gross motor function in individuals with spinal cord injury. Top Spinal Cord Inj Rehabil.

[34] Cappello L, Meyer JT, Galloway KC, Peisner JD, Granberry R, Wagner DA (2018). Assisting hand function after spinal cord injury with a fabric-based soft robotic glove. J Neuroeng Rehabil.

[35] Kapadia N, Zivanovic V, Popovic M (2013). Restoring voluntary grasping function in individuals with incomplete chronic spinal cord injury: pilot study. Top Spinal Cord Inj Rehabil.

[36] Marquez-Chin C, Marquis A, Popovic MR (2016). EEG-triggered functional electrical stimulation therapy for restoring upper limb function in chronic stroke with severe hemiplegia. Case Rep Neurol Med.

[37] Soekadar S, Witkowski M, Gómez C, Opisso E, Medina J, Cortese M (2016). Hybrid EEG/EOG-based brain/neural hand exoskeleton restores fully independent daily living activities after quadriplegia. Sci Robot.

[38] Keitll R, Granger C, Hamilton B (1987). The functional independence measure: a new tool for rehabilitstion. Adv Clin Rehabil.

[39] Anderson K, Aito S, Atkins M, Biering-Sørensen F, Charlifue S, Curt A (2008). Functional recovery measures for spinal cord injury: an evidence-based review for clinical practice and research: report of the National Institute on Disability and Rehabilitation Research spinal cord injury measures meeting. J Spinal Cord Med.

[40] Catz A, Itzkovich M, Agranov E, Ring H, Tamir A (1997). SCIM–spinal cord independence measure: a new disability scale for patients with spinal cord lesions. Spinal Cord.

[41] Catz A, Itzkovich M, Agranov E, Ring H, Tamir A (2001). The spinal cord independence measure (SCIM): sensitivity to functional changes in subgroups of spinal cord lesion patients. Spinal Cord.

[42] Demers L, Weiss-Lambrou R, Ska B (2002). The Quebec user evaluation of satisfaction with assistive technology (QUEST 2.0): an overview and recent progress. Technol Disabil.

[43] Lee S-H, Jung B-K, Park S-Y (2013). Korean translation and psychometric properties of Quebec user evaluation of satisfaction assistive technology 2.0. J Korea Acad Ind Coop Soc.

[44] Harvey L (1996). Principles of conservative management for a non-orthotic tenodesis grip in tetraplegics. J Hand Ther.

[45] Johanson ME, Murray WM (2002). The unoperated hand: the role of passive forces in hand function after tetraplegia. Hand Clin.

[46] In H, Kang BB, Sin M, Cho K-J (2015). Exo-glove: a wearable robot for the hand with a soft tendon routing system. IEEE Robot Auto Mag.

[47] Howell J. Principles and Components of Upper Limb Orthoses. Atlas of Orthoses and Assistive Devices. Amsterdam: Elsevier; 2019. p. 134–45.

[48] Yu S, Perez H, Barkas J, Mohamed M, Eldaly M, Huang T-H, et al. A Soft High Force Hand Exoskeleton for Rehabilitation and Assistance of Spinal Cord Injury and Stroke Individuals. Cornell University: arXiv preprint arXiv:1902.07112.; 2019.

[49] Yun Y, Dancausse S, Esmatloo P, Serrato A, Merring CA, Agarwal P, et al. Maestro: an EMG-driven assistive hand exoskeleton for spinal cord injury patients. Singapore: 2017 IEEE International Conference on Robotics and Automation (ICRA): IEEE; 2017. p. 2904–10.

[50] Randazzo L, Iturrate I, Perdikis S (2017). Millán JdR. Mano: a wearable hand exoskeleton for activities of daily living and neurorehabilitation. IEEE Robot Auto Lett.

[51] Heung KH, Tang ZQ, Ho L, Tung M, Li Z, Tong RK. Design of a 3D Printed Soft Robotic Hand for Stroke Rehabilitation and Daily Activities Assistance. Toronto: 2019 IEEE 16th International Conference on Rehabilitation Robotics (ICORR): IEEE; 2019. p. 65–70. 10.1109/ICORR.2019.8779449 31374608

[52] Yap HK, Khin PM, Koh TH, Sun Y, Liang X, Lim JH (2017). A fully fabric-based bidirectional soft robotic glove for assistance and rehabilitation of hand impaired patients. IEEE Robot Auto Lett.

[53] Polygerinos P, Wang Z, Galloway KC, Wood RJ, Walsh CJ (2015). Soft robotic glove for combined assistance and at-home rehabilitation. Robot Auton Syst.

[54] Edelstein JE, Bruckner J (2002). Orthotics: a comprehensive clinical approach: slack incorporated.

[55] Lenzi T, De Rossi SMM, Vitiello N, Carrozza MC (2012). Intention-based EMG control for powered exoskeletons. IEEE Trans Biomed Eng.

[56] Dellon B, Matsuoka Y (2007). Prosthetics, exoskeletons, and rehabilitation [grand challenges of robotics]. IEEE Robot Auto Mag.

[57] Fleischer C, Hommel G (2008). A human--exoskeleton interface utilizing electromyography. IEEE Trans Robot.

[58] Wolf EJ, Cruz TH, Emondi AA, Langhals NB, Naufel S, Peng GC, et al. Advanced technologies for intuitive control and sensation of prosthetics. Biomed Eng Lett. 2019;9:1–10. 10.1007/s13534-019-00127-7 PMC7046895 32175133

[59] Kang BB, Choi H, Lee H, Cho KJ (2019). Exo-Glove Poly II: A polymer-based soft wearable robot for the hand with a tendon-driven actuation system. Soft Robot.

[60] Weinstein MC, Siegel JE, Gold MR, Kamlet MS, Russell LB (1996). Recommendations of the panel on cost-effectiveness in health and medicine. JAMA.

[61] Grosse SD (2008). Assessing cost-effectiveness in healthcare: history of the $50,000 per QALY threshold. Expert Rev Pharmacoecon Outcomes Res.

[62] Federici S, Scherer M (2017). Assistive technology assessment handbook: CRC press.

[63] Kong J-Y (2016). Satisfaction evaluation for Orthoses by using QUEST. J Korea Acad Ind Coop Soc.

[64] Gul JZ, Sajid M, Rehman MM, Siddiqui GU, Shah I, Kim K-H (2018). 3D printing for soft robotics–a review. Sci Technol Adv Mater.

[65] Levi T, Bonifazi P, Massobrio P, Chiappalone M (2018). Closed-loop systems for next-generation neuroprostheses. Front Neurosci.

[66] Homberg BS, Katzschmann RK, Dogar MR, Rus D. Haptic identification of objects using a modular soft robotic gripper. Hamburg: 2015 IEEE/RSJ International Conference on Intelligent Robots and Systems (IROS): IEEE; 2015. p. 1698–705.

