# Supplementary figures and images for: Development of 3D-printed myoelectric hand orthosis for patients with spinal cord injury
Source: J Neuroeng Rehabil. 2019 Dec 30;16:162. doi: 10.1186/s12984-019-0633-6 (PMC6937865; doi:10.1186/s12984-019-0633-6)

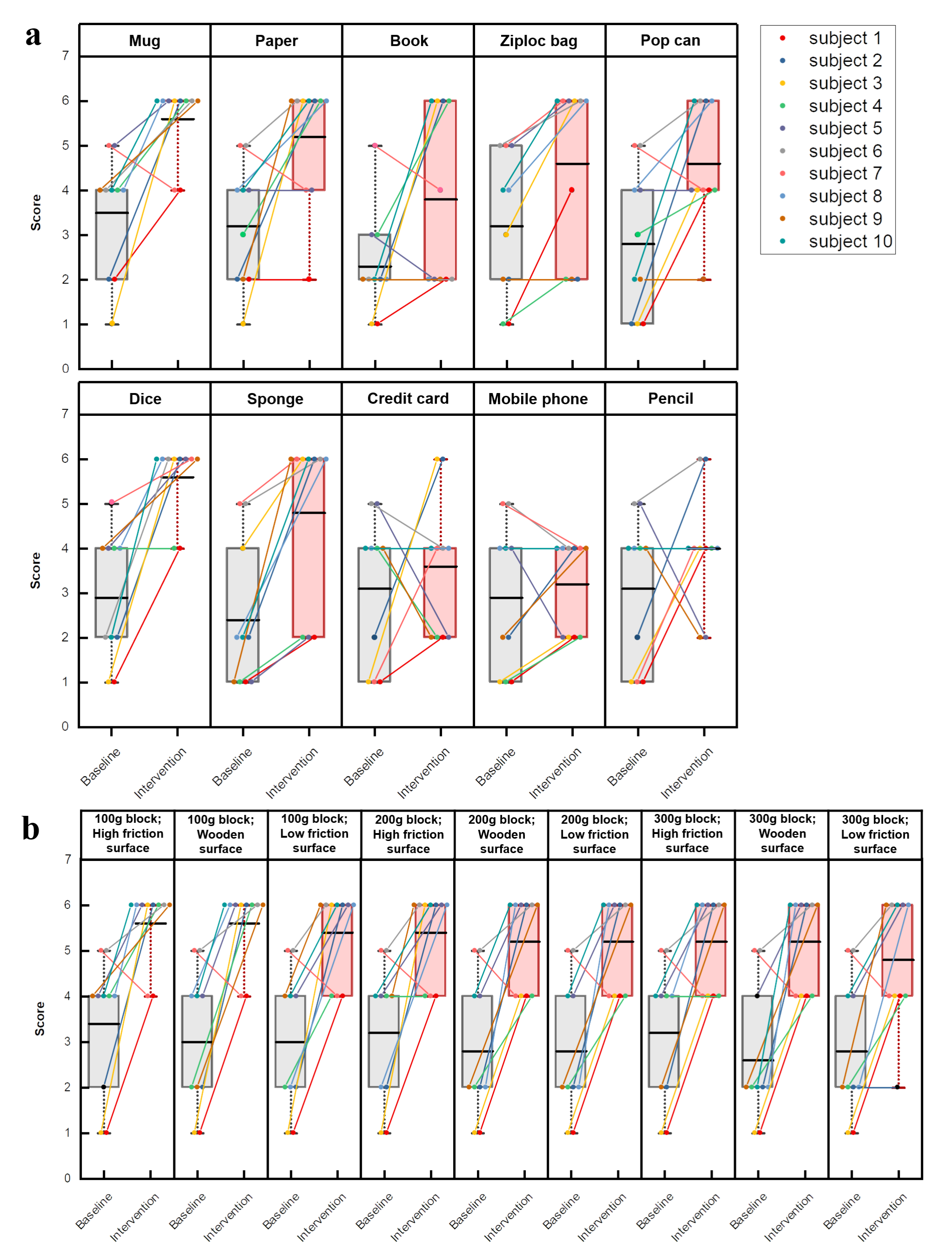

Supplement: Supplementary file 1 — Additional file 1. Distribution of the TRI-HFT scores across each individual. a Results of the TRI-HFT score in the first part. Most subjects showed improvements in hand function after wearing the orthosis but showed mixed results when dealing with small or flat objects such as a book, credit card, mobile phone, or pencil. b Results of the second part of the TRI-HFT score. The majority of subjects showed improvements in hand function with the help of the orthosis. [file 12984_2019_633_MOESM1_ESM.tif]

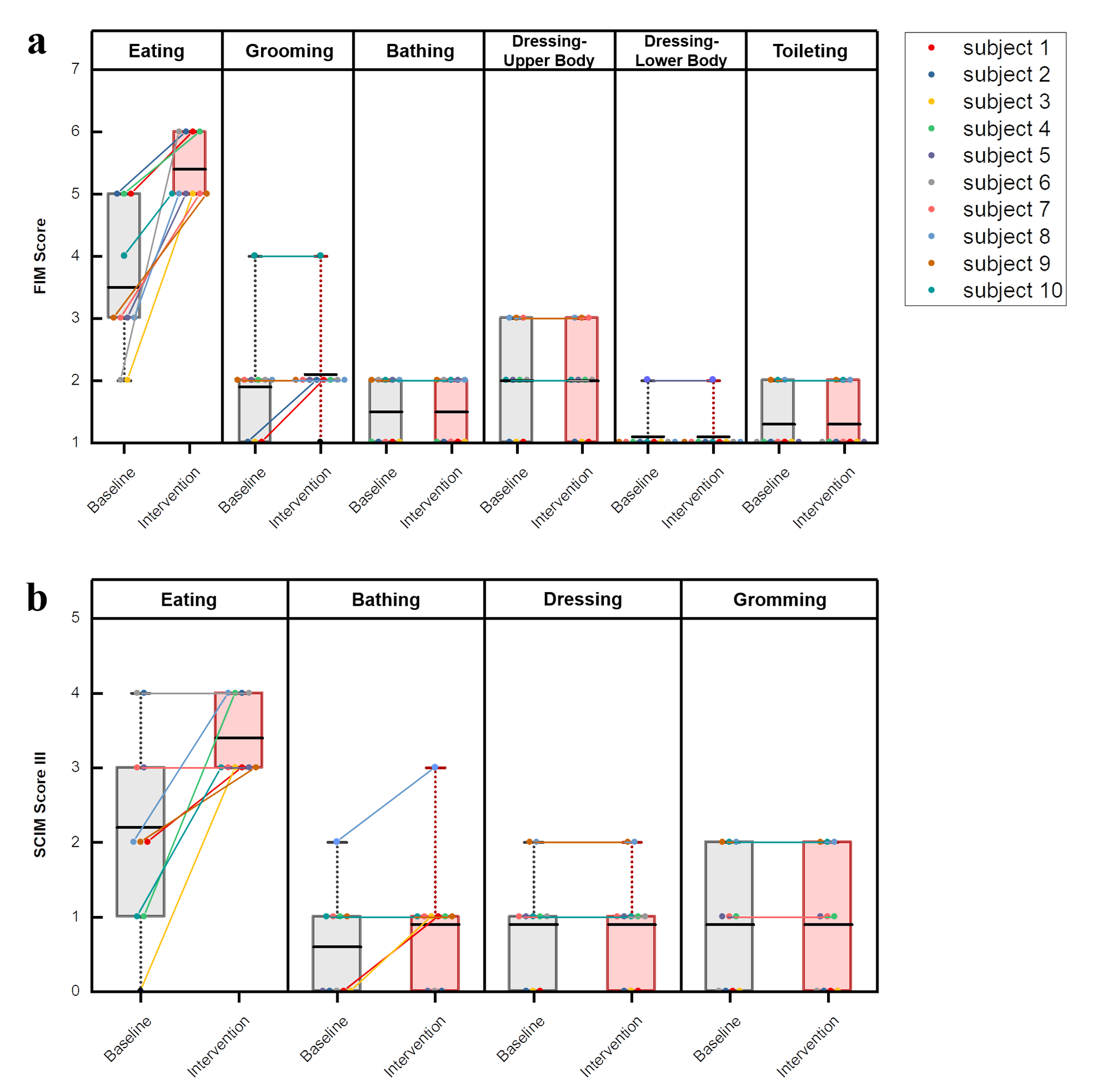

Supplement: Supplementary file 2 — Additional file 2. Distribution of ADL scores across each individual. Functional improvements were observed in the eating category. However, few improvements or no changes were observed in the remaining categories. a Results of the FIM self-care subscale. b Results of the SCIM III self-care subscale. [file 12984_2019_633_MOESM2_ESM.tif]
